# Supplementary material for: Phylogenetic analysis of plant-pathogenic and non-pathogenic Trichoderma isolates on maize from plants, soil, and commercial bio-products
Source: Appl Environ Microbiol. 2025 Feb 27;91(3):e01931-24. doi: 10.1128/aem.01931-24 (PMC11921352; doi:10.1128/aem.01931-24)
Supplement: Table A1 — Trichoderma strains obtained in the survey in Germany and from companies, universities, and research institu-tions, used in the phylogenetic analyses and the pathogenicity testing. [file aem.01931-24-s0001.docx]

RESEARCH ARTICLE

**Phylogenetic analysis of pathogenic and non-pathogenic *Trichoderma* isolates from plants, soil and commercial bio products**

Short titel: Phylogenetic analysis of pathogenic and non-pathogenic Trichoderma isolates

**Annette Pfordt ^1^, Clovis Douanla-Meli ^2^, Bernhard Schäfer^2^, Gritta Schrader^2^, Eike Tannen ^1^, Madhav Jatin Chandarana^1^, Andreas von Tiedemann ^1^**

^1^ Plant Pathology and Crop Protection, University of Goettingen, Goettingen, Germany

^2^ Julius Kühn-Institut (JKI) - Federal Research Centre for Cultivated Plants, Institute for National and Inter-national Plant Health, Germany

* Correspondence: annette.pfordt@uni-goettingen.de

**Appendix A**

**Table A1.** *Trichoderma* strains obtained in the survey in Germany and from companies, universities and research institu-tions, used in the phylogenetic analyses and the pathogenicity testing

| **Species** | **Code** | **Year** | **Geographic origin** | **Host/substrat** | **GenBank** | |
| --- | --- | --- | --- | --- | --- | --- |
|  |  |  |  |  | ***TEF1****-α* | ***RPB2*** |
| *T. afarasin* | DIS 377A | - | Cameroon | Stem, endophyte | FJ463322 | FJ442799 |
| *T. afarasin* | Dis 314F | - | Cameroon | Stem, endophyte | FJ463400 | FJ442778 |
| *T. afroharzianum* | AP18TRI1 | 2018 | Croix de Pardies (FR) | Maize | PQ558165 | PQ629815 |
| *T. afroharzianum* | AP18TRI2 | 2018 | Kuenzing (DE) | Maize | PQ558166 | PQ629816 |
| *T. afroharzianum* | AP18TRI3 | 2018 | Pocking (DE) | Maize | PQ558167 | PQ629817 |
| *T. afroharzianum* | AP19TRI5 | 2019 | Bernburg (DE) | Maize | PQ558170 | PQ629820 |
| *T. afroharzianum* | CBS 124620 | - | - | - | PQ558230 | PQ629874 |
| *T. afroharzianum* | KG10 | 2018 | Kavadarci (MKD) | *P. ostreatus* | PQ558233 | PQ629877 |
| *T. afroharzianum* | KG13 | 2018 | Kavadarci (MKD) | *P. ostreatus* | PQ558234 | PQ629878 |
| *T. afroharzianum* | MRI349 | - | - | - | PQ558235 | PQ629879 |
| *T. afroharzianum* | DISAFATS-1 | 2021 | Carmagnola (IT) | Maize | PQ558263 | PQ629907 |
| *T. afroharzianum* | AP22TRI81_1 | 2022 | Rustenhart (DE) | Maize | PQ558223 | PQ629867 |
| *T. afroharzianum* | AP22TRI84 | 2022 | Rustenhart (DE) | Maize | PQ558224 | PQ629868 |
| *T. afroharzianum* | AP22TRI85 | 2022 | Rustenhart (DE) | Maize | PQ558225 | PQ629869 |
| *T. afroharzianum* | AP22TRI99 | 2022 | Bevern (DE) | Soil | PQ558267 | PQ629911 |
| *T. afroharzianum* | AP22TRI110 | 2022 | Hasloch 1 (DE) | Soil | PQ558183 | PQ629833 |
| *T. afroharzianum* | AP22TRI111 | 2022 | Hasloch 2 (DE) | Soil | PQ558268 | PQ629912 |
| *T. afroharzianum* | AP22TRI113 | 2022 | Bevern Hoeltje (DE) | Soil | PQ558185 | PQ629835 |
| *T. afroharzianum* | AP22TRI114 | 2022 | Bevern Ricke (DE) | Soil | PQ558186 | PQ629836 |
| *T. afroharzianum* | AP22TRI130 | 2022 | Oberpframmern (DE) | Soil | PQ558201 | PQ629848 |
| *T. afroharzianum* | AP22TRI131 | 2022 | Oberpframmern (DE) | Soil | PQ558202 | PQ629849 |
| *T. afroharzianum* | AP22TRI134 | 2022 | Oberpframmern (DE) | Soil | PQ558205 | PQ629850 |
| *T. afroharzianum* | AP22TRI136 | 2022 | Oberpframmern (DE) | Soil | PQ558207 | PQ629852 |
| *T. afroharzianum* | AP22TRI137 | 2022 | Rustenhart (DE) | Soil | PQ558208 | PQ629853 |
| *T. afroharzianum* | AP22TRI148 | 2022 | Haßloch 2 (DE) | Soil | PQ558269 | PQ629913 |
| *T. afroharzianum*^T^ | GJS 04 186 | - | Peru | *Moniliophthora roreri* | FJ463301 | FJ442691 |
| *T. afroharzianum* | LESF229 | - | - | Soil | KT279013 | KT278945 |
| *T. afroharzianum* | Tri-1 | - | - | - | OP102131 | OP102132 |
| *T. arundinaceum* | TR1 | 2016 | Leskovac (SRB) | Tomato, root | - | PQ629917 |
| *T. arundinaceum* | AP22TRI129 | 2022 | Nossen (DE) | Soil | PQ558200 | - |
| *T. arundinaceum* | ATCC 90237 | - | Namibia | Soil | EU338291 | EU338326 |
| *T. arundinaceum* | GJS 05-183 | - | Iran | - | EU338274 | EU338302 |
| *T. asperelloides* | **HOHTR22** | - | - | - | PQ558256 | PQ629900 |
| *T. asperelloides* | GJS 04-116 | - | Vietnam | Soil | GU248412 | GU248411 |
| *T. asperelloides* | NT8 | - | Nepal | Soil | MW408215 | MZ355338 |
| *T. asperellum* | TR2 | 2015 | Topola (SRB) | Apricot, fruit | PQ558255 | PQ629899 |
| *T. asperellum* | TR4 | 2015 | Topola (SRB) | Apricot, fruit | PQ558266 | PQ629910 |
| *T. asperellum* | XILONT34 | - |  | - | PQ558265 | PQ629909 |
| *T. asperellum* | ABITEP02 | - |  | - | PQ558164 | PQ629814 |
| *T. asperellum* | AP22TRI100 | 2022 | Bevern Forst (DE) | Soil | PQ558173 | PQ629823 |
| *T. asperellum* | AP22TRI142 | 2022 | Rustenhart (DE) | Soil | PQ629875 | PQ629922 |
| *T. asperellum* | CGMCC 6422 | - | China | - | KF425756 | KF425755 |
| *T. asperellum* | GJS 06-314 | - | Ecuador | Plantain | GU198292 | GU198278 |
| *T. atrobrunneum* | T54 | 2008 | Kula (SRB) | *A. bisporus* fb | PQ558272 | PQ629914 |
| *T. atrobrunneum* | AP22TRI149 | 2022 | Bevern Höltje (DE) | Soil | PQ558218 | PQ629862 |
| *T. atrobrunneum* | GJS 04-67 | - | Italy | Soil | FJ463360 | FJ442724 |
| *T. atrobrunneum* | SZMC 26673 | - | North Macedonia | *P. ostreatus* | MZ773434 | MZ773413 |
| *T. atroviride* | IPP0316 | 1976 | - | Food | PQ558231 |  |
| *T. atroviride* | T33 | 2006 | Zemun Polje (SRB) | *A. bisporus fb* | PQ558243 | PQ629887 |
| *T. atroviride* | T60 | 2008 | Zemun (SRB) | *A. bisporus fb* | PQ558250 | PQ629894 |
| *T. atroviride* | TR8 | 2019 | Belgrade (SRB) | Tomato, root | PQ558261 | PQ629905 |
| *T. atroviride* | TR10 | 2019 | Leskovac (SRB) | Tomato, rhizosphere | PQ558253 | PQ629897 |
| *T. atroviride* | HOHT20 | - | - | - | PQ558240 | PQ629884 |
| *T. atroviride* | AP22TRI102 | 2022 | Bevern Lindenbusch (DE) | Soil | PQ558175 | PQ629825 |
| *T. atroviride* | AP22TRI103 | 2022 | Göttingen Königsbühl (DE) | Soil | PQ558176 | PQ629826 |
| *T. atroviride* | AP22TRI107 | 2022 | Pocking (DE) | Soil | PQ558180 | PQ629830 |
| *T. atroviride* | AP22TRI109 | 2022 | Pocking (DE) | Soil | PQ558182 | PQ629832 |
| *T. atroviride* | AP22TRI115 | 2022 | Granswang KGW 3 (DE) | Soil | PQ558187 | PQ629837 |
| *T. atroviride* | AP22TRI116 | 2022 | Granswang KGW 3 (DE) | Soil | PQ558188 | PQ629838 |
| *T. atroviride* | AP22TRI117 | 2022 | Granswang KGW 3 (DE) | Soil | PQ558189 | - |
| *T. atroviride* | AP22TRI119 | 2022 | Granswang KGW 3 (DE) | Soil | PQ558191 | PQ629840 |
| *T. atroviride* | AP22TRI128 | 2022 | Kuenzing (DE) | Soil | PQ558199 | PQ629847 |
| *T. atroviride* | AP22TRI139 | 2022 | Rustenhart (DE) | Soil | PQ558210 | PQ629855 |
| *T. atroviride* | CBS 119499 | - | - | - | FJ860611 | FJ860518 |
| *T. atroviride* | CBS 141.95 | 2003 | Slovenia | Beetle gallery | AY376051 | EU341801 |
| *T. auriculariae*^T^ | JZBQT1Z7 | - | China | - | ON649896 | ON649949 |
| *T. auriculariae* | JZBQT1Z8 | - | China | - | ON649897 | ON649950 |
| *T. azevodoi* | IPP0318 | 1992 | Ban Vieng (CN) | Soil | PQ629921 | PQ629920 |
| *T. azevodoi* | IPP0320 | 1992 | Chiang Mai (CN) | Soil | PQ558271 | PQ629876 |
| *T. azevodoi* | AP19TRI6 | 2019 | KWS (DE) | Maize | PQ558171 | PQ629923 |
| *T. azevodoi* | AP19TRI7 | 2019 | Grucking (DE) | Maize | - | PQ629915 |
| *T. azedevoi* | AP19TRI8 | 2019 | Großumstadt (DE) | Maize | PQ558172 | PQ629822 |
| *T. azedevoi* | AP19TRI10 | 2019 | Großumstadt (DE) | Maize | PQ558168 | PQ629818 |
| *T. azevedoi* | AP19TRI11 | 2019 | Pfaffenhofen (DE) | Maize | PQ558169 | PQ629819 |
| *T. azevodoi* | CEN1403 | - | Brazil | Soil | MK696638 | MK696800 |
| *T. azevodoi* | CEN1422 | - | Brazil | Soil | MK696660 | MK696821 |
| *T. azevodoi* | CEN1423 | - | Brazil | Soil | MK696661 | MK696822 |
| *T. bannaense*^T^ | HMAS 248840 | - | China | - | KY688037 | KY687979 |
| *T. bannaense* | HMAS 248865 | - | China | - | KY688038 | KY688003 |
| *T. breve*^T^ | HMAS 248844 | - | China | - | KY688045 | KY687983 |
| *T. breve* | HMAS 248845 | - | China | - | KY688046 | KY687984 |
| *T. brevicompactum* | AP22TRI140 | 2022 | Rustenhart (DE) | Soil | PQ558211 | - |
| *T. brevicompactum* | CBS 112444 | - | Mexico | Soil | EU338296 | EU338314 |
| *T. brevicompactum* | CBS 112447 | - | Mexico | Soil | EU338300 | EU338318 |
| *T. camerunense* | Vimi-17 0025 | - | Brazil | Termite nest | MZ675902 | MZ675866 |
| *T. camerunense* | Vimi-17 0034 | - | Brazil | Termite nest | MZ675908 | MZ675862 |
| *T. cerinum* | AP22TRI97 | 2022 | Göttingen (DE) | Soil | PQ558228 | PQ629872 |
| *T. cerinum* | AP22TRI112 | 2022 | Hasloch 3 (DE) | Soil | PQ558184 | PQ629834 |
| *T. cerinum* | AP22TRI126 | 2022 | Nossen (DE) | Soil | PQ558197 | PQ629846 |
| *T. cerinum* | AP22TRI138 | 2022 | Rustenhart (DE) | Soil | PQ558209 | PQ629854 |
| *T. cerinum* | AP22TRI150 | 2022 | Bevern Höltje (DE) | Soil | PQ558219 | PQ629863 |
| *T. cerinum* | 31.24.06.1 | - | Poland | - | KP008913 | KP009174 |
| *T. cerinum* | S357 | - | France | - | KF134797 | KF134788 |
| *T. endophyticum* | Dis 221E | - | Ecuador | *Theobroma gileri* | FJ463316 | FJ442775 |
| *T. endophyticum* | MMSRG85 | - | Brazil | *Botrylloides giganteus* | OQ291284 | OQ291285 |
| *T. guizhouense* | T10 | 2006 | Požarevac (SRB) | *A. bisporus* fruiting body (fb) | KC555182 | PQ629882 |
| *T. guizhouense* | T52 | 2008 | Zemun (SRB) | *A. bisporus fb* | KC555177 | PQ629890 |
| *T. guizhouense* | T57 | 2008 | Ugrinovci (SRB) | *L. edodes fb* | MT876593] | PQ629891 |
| *T. guizhouense* | T59 | 2008 | Ugrinovci (SRB) | *L. edodes fb* | MT876595 | PQ629893 |
| *T. guizhouense* | T2 | 2017 | Kruševac (SRB) | Strawberry | PQ558239 | PQ629883 |
| *T. guizhouense* | T3 | 2017 | Leskovac (SRB) | Tomato, rhizosphere | PQ558242 | PQ629886 |
| *T. guizhouense* | T4 | 2017 | Čelarevo (SRB) | Cherry, rhizosphere | PQ558245 | PQ629889 |
| *T. guizhouense* | TR5 | 2017 | Užice (SRB) | Tomato, rhizosphere | PQ558258 | PQ629902 |
| *T. guizhouense* | TRICHOSTAR T58 | - |  | Biostimulant | PQ558248 | PQ629892 |
| *T. guizhouense* | E39 | - | Kenya | *Coffea* sp. | MK044084 | MK044176 |
| *T. guizhouense* | S548 | - | Spain | - | KJ665507 | KJ665031 |
| *T. guizhouense* | S628 | - | Greece | - | KJ665511 | KJ665273 |
| *T. guizhouense* | SZMC 22514 | - | Croatia | *P. ostreatus* | MZ773448 | MZ773427 |
| *T. guizhouense* | DIS 314D | - | Cameroon | Stem | FJ463355 | FJ442719 |
| *T. hamatum* | AP22TRI152 | 2022 | Oberpframmern (DE) | Soil | PQ558221 | PQ629865 |
| *T. hamatum* | CBS:132565 |  | France |  | KJ665514 | KJ665275 |
| *T. hamatum* | Dis 216d | 2022 | Ecuador | Soil | EU856313 | FJ150778 |
| *T. harzianum* | T1 | 2017 | Kruševac (SRB) | Strawberry | PQ558237 | PQ629881 |
| *T. harzianum* | TR7 | 2016 | Požega (SRB) | Tomato, rhizosphere | PQ558260 | PQ629904 |
| *T. harzianum* | AP22TRI104 | 2022 | Göttingen Königsbühl (DE) | Soil | PQ558177 | PQ629827 |
| *T. harzianum* | AP22TRI106 | 2022 | Pocking (DE) | Soil | PQ558179 | PQ629829 |
| *T. harzianum* | AP22TRI123 | 2022 | Granswang KGW 4 (DE) | Soil | PQ558195 | PQ629844 |
| *T. harzianum* | AP22TRI135 | 2022 | Oberpframmern (DE) | Soil | PQ558206 | PQ629851 |
| *T. harzianum* | AP22TRI151 | 2022 | Oberpframmern (DE) | Soil | PQ558220 | PQ629864 |
| *T. harzianum* | AP22TRI153 | 2022 | Bevern Ricke (DE) | Soil | PQ558222 | PQ629866 |
| *T. harzianum* | CBS 226.95^N^ | - | - | - | MH874152 | AF545549 |
| *T. harzianum* | DIS 314D | - | Cameroon | Stem | FJ463355 | FJ442719 |
| *T. harzianum* | GJS 04-71 | - | Italy | *Castanea sativa* | FJ463396 | FJ442779 |
| *T. harzianum* | GJS 05-107 | - | Italy | *Ricinus communis* | FJ463329 | FJ442708 |
| *T. inhamatum*^T^ | CBS 273.78 | - | Colombia | Soil maize field | AF348099 | FJ442725 |
| *T. koningii* | T39 | 2007 | Veliko Gradište (SRB) | *A. bisporus fb* | PQ558244 | PQ629888 |
| *T. koningii* | GJS 90-18 |  | USA:WI | Burned wood | DQ289007 | EU248600 |
| *T. koningiopsis* | AP22TRI98 | 2022 | Göttingen (DE) | Soil | PQ558229 | PQ629873 |
| *T. koningiopsis* | AP22TRI105 | 2022 | Göttingen (DE) | Soil | PQ558178 | PQ629828 |
| *T. koningiopsis* | GJS 93-20 | - | Cuba | Branch | DQ284966 | EU241506 |
| *T. lentiforme* | DIS 218E | 2003 | Ecuador | *Theobroma gileri* | FJ463310 | FJ442793 |
| *T. lentiforme* | DIS 173F | 2003 | Brazil | *Theobroma* sp. | FJ463347 | FJ442787 |
| *T. linzhiense* | HMAS 248874 | 2016 | China, Tibet | Soil | KY688048 | KY688011 |
| *T. linzhiense*^T^ | HMAS 248846 | 2015 | China, Tibet | Soil | KY688047 | KY687985 |
| *T. neocrassum*^T^ | DAOM 164916 | 2014 | Canada | *Picea* sp. wood | EU80048 | KJ842185 |
| *T. neocrassum* | GJS 95-157 | - | New York, USA | Decorticated wood | AF534602 | AF545543 |
| *T. paratroviride* | VINTEC_SC1 | - | - | - | PQ558236 | PQ629880 |
| *T. paratroviride* | a131 | 2022 | China | Maize | ON934351 | ON934386 |
| *T. paratroviride*^T^ | CBS:136489 | 2010 | Spain | *Phillyrea angulistifolia* | KJ665627 | KJ665321 |
| *T. paraviridescens* | HOHUHBot | - | - | - | PQ558264 | PQ629908 |
| *T. paraviridescens* | CBS 274.79 | - | Austria | - | DQ307513 | EU252010 |
| *T. paraviridescens* | S122 | - | Italy | - | KC285671 | KC285764 |
| *T. peberdyi* | AP22TRI96 | 2022 | Bevern (DE) | Soil | PQ558227 | PQ629871 |
| *T. peberdyi* | AP22TRI101 | 2022 | Bevern Forst (DE) | Soil | PQ558174 | PQ629824 |
| *T. peberdyi* | AP22TRI120 | 2022 | Granswang (DE) | Soil | PQ558192 | PQ629841 |
| *T. peberdyi* | AP22TRI122 | 2022 | Granswang (DE) | Soil | PQ558194 | PQ629843 |
| *T. peberdyi* | AP22TRI124 | 2022 | Granswang (DE) | Soil | PQ558196 | PQ629845 |
| *T. peberdyi* | AP22TRI143 | 2022 | Haßloch 1 (DE) | Soil | PQ558213 | PQ629857 |
| *T. peberdyi* | AP22TRI144 | 2022 | Haßloch 1 (DE) | Soil | PQ558214 | PQ629858 |
| *T. peberdyi* | AP22TRI145 | 2022 | Haßloch 1 (DE) | Soil | PQ558215 | PQ629859 |
| *T. peberdyi* | AP22TRI146 | 2022 | Haßloch 1 (DE) | Soil | PQ558216 | PQ629860 |
| *T. peberdyi* | AP22TRI147 | 2022 | Haßloch 2 (DE) | Soil | PQ558217 | PQ629861 |
| *T. peberdyi* | CEN1387 | 2015 | Brazil | Soil | MK696619 | MK696781 |
| *T. peberdyi* | CEN1388 | 2015 | Bzazil | Soil | MK696620 | MK696782 |
| *T. petersenii* | CBS 119507 | 2003 | Austria | *Salix caprea* | FJ860670 | FJ860568 |
| *T. petersenii* | GJS 04-164 | - | Tennessee, USA | Decorticated wood | DQ289004 | FJ442783 |
| *T. polypori*^T^ | HMAS 248855 | 2015 | China, Hunan | Dried polypore | KY688058 | KY687994 |
| *T. polypori* | HMAS 248861 | 2015 | China, Hunan | Polypore | KY688059 | KY688000 |
| *T. protrudens* | DIS 119F | - | India | *Theobroma cacao* | EU338289 | EU338322 |
| *T. protrudensi*^T^ | CBS 121320 | - | India | *Theobroma cacao* | - | OK813901 |
| *T. pseudopyramidale*^T^ | E720 | - | Ethiopia | *Coffea arabica* | MK044131 | MK044224 |
| *T. pseudopyramidale* | M307 | - | Ethiopia | *Coffea arabica* | MK044162 | MK044225 |
| *T. pyramidale*^T^ | CBS 135574 | - | Italy | - | KJ665699 | KJ665334 |
| *T. pyramidale* | S573 | - | Italy | - | KJ665698 | - |
| *T. rifaii* | Dis 337F | - | Panama | Stem, endophyte | FJ463321 | FJ442720 |
| *T. rifaii*^T^ | DIS 355B | - | Ecuador | *Theobroma gileri* | FJ463324 | - |
| *T. simmonsii* | T64 | 2009 | Zemun (SRB) | *A. bisporus fb* | PQ558251 | PQ629895 |
| *T. simmonsii* | TR9 | 2018 | Leskovac (SRB) | Tomato, rhizosphere | PQ558262 | PQ629906 |
| *T. simmonsii* | BIOHEALTH_T50 | - | - | - | PQ629919 | PQ629918 |
| *T. simmonsii* | GJS 90-127 | - | North Carolina, USA | Wood and fungus | - | FJ442798 |
| *T. simmonsii* | S7 | - | Italy | - | KJ665719 | KJ665337 |
| *T. simmonsii* | SZMC 26671 | - | Serbia | *Pleurotus ostreatus* | MZ773436 | MZ773415 |
| *Trichoderma* sp1*.* | TR6 | 2010 | Belgrade (SRB) | Orchids, rhizosphere | PQ558259 | PQ629903 |
| *Trichoderma* sp1*.* | TR11 | 2011 | Belgrade (SRB) | Orchids, leaf | PQ558254 | PQ629898 |
| *Trichoderma* sp2. | TERRAX_T720 | - | - | - | PQ558252 | PQ629896 |
| *Trichoderma* sp2. | AP22TRI118 | 2022 | Granswang (DE) | Soil | PQ558190 | PQ629839 |
| *T. tomentosum* | CBS 120637 | - | - | - | FJ860629 | FJ860532 |
| *T. tomentosum*^T^ | DAOM 178713A | - | - | - | EU279969 | AF545557 |
| *T. velutinum* | AP22TRI95 | 2022 | Bevern (DE) | Soil | PQ558226 | PQ629870 |
| *T. velutinum* | AP22TRI108 | 2022 | Pocking (DE) | Soil | PQ558181 | PQ629831 |
| *T. velutinum* | AP22TRI121 | 2022 | Granswang (DE) | Soil | PQ558193 | PQ629842 |
| *T. velutinum* | CPK 298 | - | Nepal | - | KF134794 | KJ665769 |
| *T. velutinum*^T^ | DAOM 230013 | - | Nepal | - | AY605803 | JN133569 |
| *T. virens* | AP22TRI141 | 2022 | Rustenhart (DE) | Soil | PQ558212 | PQ629856 |
| *T. virens* | DIS 162 | - | Costa Rica | *Theobroma cacao* | FJ463367 | FJ442696 |
| *T. virens* | E174 | - | Cameroon | *Coffea brevipes* | MK044087 | MK044180 |
| *T. virens* | GJS 01-287 | - | Cote d'Ivoire | Soil | AY750894 | EU341804 |
