# Supplementary material for: Phylogenetic analysis of plant-pathogenic and non-pathogenic Trichoderma isolates on maize from plants, soil, and commercial bio-products
Source: Appl Environ Microbiol. 2025 Feb 27;91(3):e01931-24. doi: 10.1128/aem.01931-24 (PMC11921352; doi:10.1128/aem.01931-24)
Supplement: Table S1 and Fig. S1 — Molecular identification of Trichoderma strains used in this study, and phylogenetic tree based on maximum likelihood analysis of TEF1-α and RPB2 sequence data set. [file aem.01931-24-s0002.docx]

**Table S1.** Molecular identification of *Trichoderma* strains used in this study based on the protocol proposed by Cai and Druzhinina (2021) by estimating the pairwise similarity between the ITS, TEF1 and RPB2 sequences obtained and the sequences of reference (type) material.

|  | **Similarity standard** | | |  | | | |
| --- | --- | --- | --- | --- | --- | --- | --- |
|  | **Genus** | **Species** | **Species** |  |  |  |  |
|  | **ITS** | **RPB2** | **TEF1** |  |  |  |  |
|  | ≥76% | ≥99% | ≥97% |  |  |  |  |
|  | **Similarity calculated** | | | **Identified species** | **Verification** | | |
| **Strains** | ≥76% | ≥99% | ≥97% |  | **Phylogenetic concordance of RPB2 and TEF1** | **Status of**  **species**  **identification** | **Reference sequence/isolate** |
| AP18TRI1, AP18TRI2, AP18TRI3,  AP19TRI5, AP20TRI15, AP22TRI81-1, AP22TRI84, AP22TRI85, AP22TRI99, AP22TRI110, AP22TRI111, AP22TRI113, AP22TRI114, AP22TRI130, AP22TRI131, AP22TRI134, AP22TRI136, AP22TRI137, AP22TRI148, KG10, KG13, MRI349, TS-1 | *Trichoderma* | *T. afroharzianum* | *T. afroharzianum* | *T. afroharzianum* | Yes | Unambiguous | *T. afroharzianum* CBS 124620 |
| AP22TRI129, TR1 | *Trichoderma* | *T. arundinaceum* | *T. arundinaceum* | *T. arundinaceum* | Yes | Unambiguous | *T. arundinaceum* GJS 05-180 |
| HOHTR22 | *Trichoderma* | *T. asperelloides* | *T. asperelloides* | *T. asperelloides* | Yes | Unambiguous | *T.* asperelloides  GJS 04-111 |
| ABITEP02, AP22TRI100, AP22TRI142, TR2, TR3, TR4, XILONTT34 | *Trichoderma* | *T. asperellum* | *T. asperellum* | *T. asperellum* | Yes | Unambiguous | *T. asperellum*  CBS 433.97 |
| AP22TRI149, T54 |  | *T. atrobrunneum* | *T. atrobrunneum* | *T. atrobrunneum* | Yes | Unambiguous | CBS 548.92  *T. atrobrunneum* |
| AP22TRI102, AP22TRI103, AP22TRI107,  AP22TRI109,  AP22TRI113,  AP22TRI115  AP22TRI116,  AP22TRI117, AP22TRI119, AP22TRI128, AP22TRI139, IPPO316,  HOHT20, T33, T60, TR10 | *Trichoderma* | *T. atroviridae* | *T. atroviridae* | *T. atroviridae* | Yes | Unambiguous |  |
| AP19TRI6, AP19TRI7, AP19TRI8, AP19TRI10, AP19TRI10, IPPO318, IPPO320 | *Trichoderma* | *T.* *azevedoi* | *T. azevedoi* | *T. azevedoi* | Yes | Unambiguous | *T. azevedoi* CEN1422 |
| AP22TRI140 | *Trichoderma* | *T.* *brevicompactum* | *T.* *brevicompactum* | *T.* *brevicompactum* | Yes | Unambiguous | *T.* *brevicompactum*  CBS 109720 |
| AP22TRI97, AP22TRI112, AP22TRI138, AP22TRI126, AP22TRI150 | *Trichoderma* | *T. cerinum* | *T. cerinum* | *T. cerinum* | Yes | Unambiguous | *T. cerinum*  ATCC MYA-4840 |
| T2, T3, T4, T10, T52, T57, T58, T59, TR3, TR5 | *Trichoderma* | *T. guizhouense* | *T. guizhouense* | *T. guizhouense* | Yes | Unambiguous | *T. guizhouense*  CBS 131803 |
| AP22TRI152 | *Trichoderma* | *T. hamatum* | *T. hamatum* | *T. hamatum* | Yes | Unambiguous | *T. hamatum*  DAOM 167057 |
| AP22TRI104, AP22TRI106, AP22TRI123, AP22TRI135, AP22TRI151, AP22TRI153, T1, TR7 | *Trichoderma* | *T. harzianum* | *T. harzianum* | *T. harzianum* | Yes | Unambiguous | *T. harzianum*  CBS 226.95 |
| T39 | *Trichoderma* | *T. koningii* | *T. koningii* | *T. koningii* | Yes | Unambiguous | *T. koningii*  ATCC 64262 |
| AP22TRI98, AP22TRI105 | *Trichoderma* | *T. koningiopsis* | *T. koningiopsis* | *T. koningiopsis* | Yes | Unambiguous | *T. koningiopsis*  CBS 119075 |
| VINTEC_SC1 | *Trichoderma* | *T. paratroviride* | *T. paratroviride* | *T. paratroviride* | Yes | Unambiguous | *T. paratroviride*  CBS 136489 |
| HOHUHBot | *Trichoderma* | *T. paraviridescens* | *T. paraviridescens* | *T. paraviridescens* | Yes | Unambiguous | *T. paraviridescens*  CBS 119321 |
| AP22TRI96, AP22TRI101, AP22TRI120, AP22TRI122, AP22TRI124, AP22TRI143, AP22TRI144, AP22TRI145, AP22TRI146, AP22TRI147 | *Trichoderma* | *T. peberdyi* | *T. peberdyi* | *T. peberdyi* | Yes | Unambiguous | T. *peberdyi*  CEN 1426 |
| BIOHEALTH_T5, TRICHOSAN, T64, TR9 | *Trichoderma* | *T.* *simmonsii* | *T.* *simmonsii* | *T.* *simmonsii* | Yes | Unambiguous | *T.* *simmonsii*  CBS 130431 |
| AP22TRI95, AP22TRI108, AP22TRI121 | *Trichoderma* | *T. velutinum* | *T. velutinum* | *T. velutinum* | Yes | Unambiguous | *T. velutinum*  ATCC MYA-4841 |
| AP22TRI141 | *Trichoderma* | *T. virens* | *T. virens* | *T. virens* | Yes | Unambiguous | *T. virens*  CBS 109339 |
| TR6, TR11 | *Trichoderma* | *T. auriculariae* | *T. anaharzianum* | T. sp.  New species close to *T. auriclariae* | yes | Unambiguous | *-* |
| AP22TRI118, TERRAXT 720 | *Trichoderma* | - | *T. atrobrunneum* | T. sp.  New species close to  *T. atrobrunneum* | No | Ambiguous | - |

NB. All strains unambiguously identified and assigned to a known *Trichoderma* species met the condition: *Trichoderma*[ITS_76_ ] ~ sp∃!(rpb2_99_ ≅ tef1_97_)

Where “*Trichoderma”* means the genus *Trichoderma*, “sp” means a species, “~” indicates an agreement between ITS and other loci, “≅” refers to the concordance between “*rpb2”* and “*tef1”*, and “∃!” indicates the uniqueness of the condition (Cai and Druzhinina, 2021).


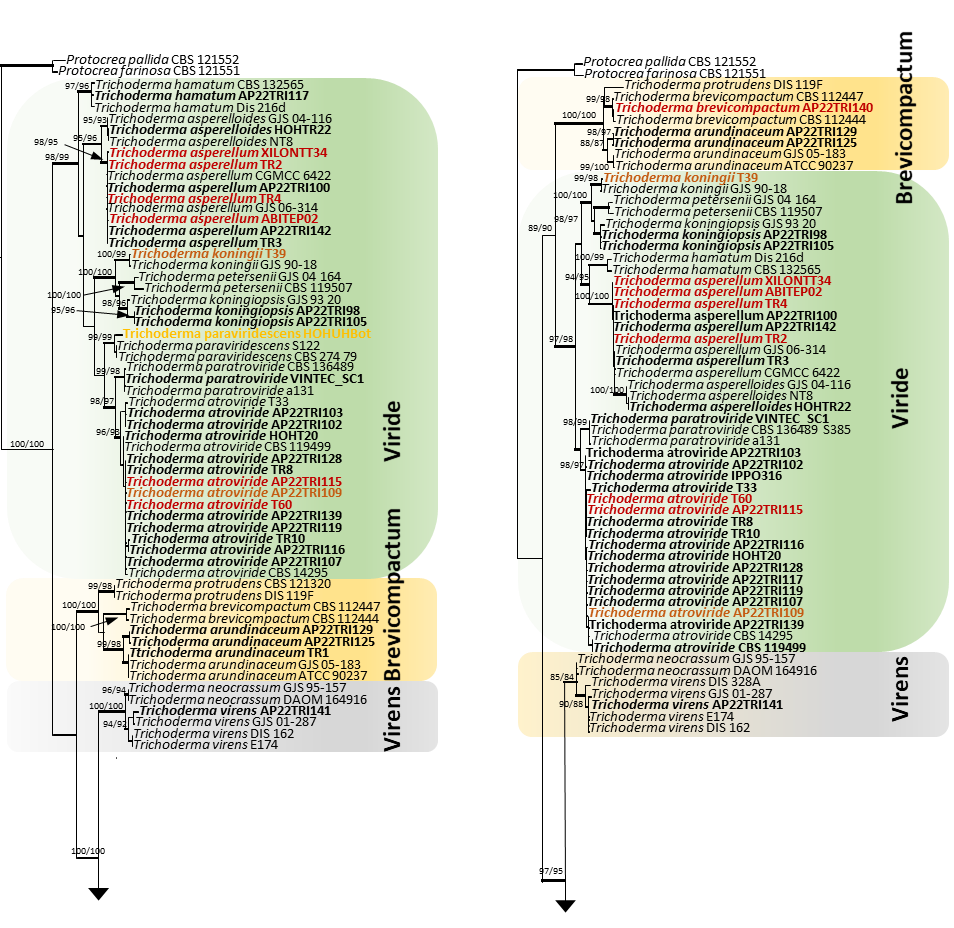


**A B**

**Figure S1.** Phylogenetic tree based on Maximum Likelihood analysis of TEF1-α (A) and RPB2 (B) sequence dataset. Bootstrap values higher than 70 % from MP (left) and RAxML (right) analyses are given above the nodes. Thickened lines represent branches with a Bayesian posterior probability greater than 0.95. Isolates analyzed and tested for pathogenicity on maize in this study are in bold. Pathogenetic isolates are in bold color with red for high, brown for moderate and orange for weak disease severity. *Protocrea farinosa* (CBS 121551) and *Protocrea* *pallida* (CBS 121552) were used as outgroup.

**Figure S1 (continued)**


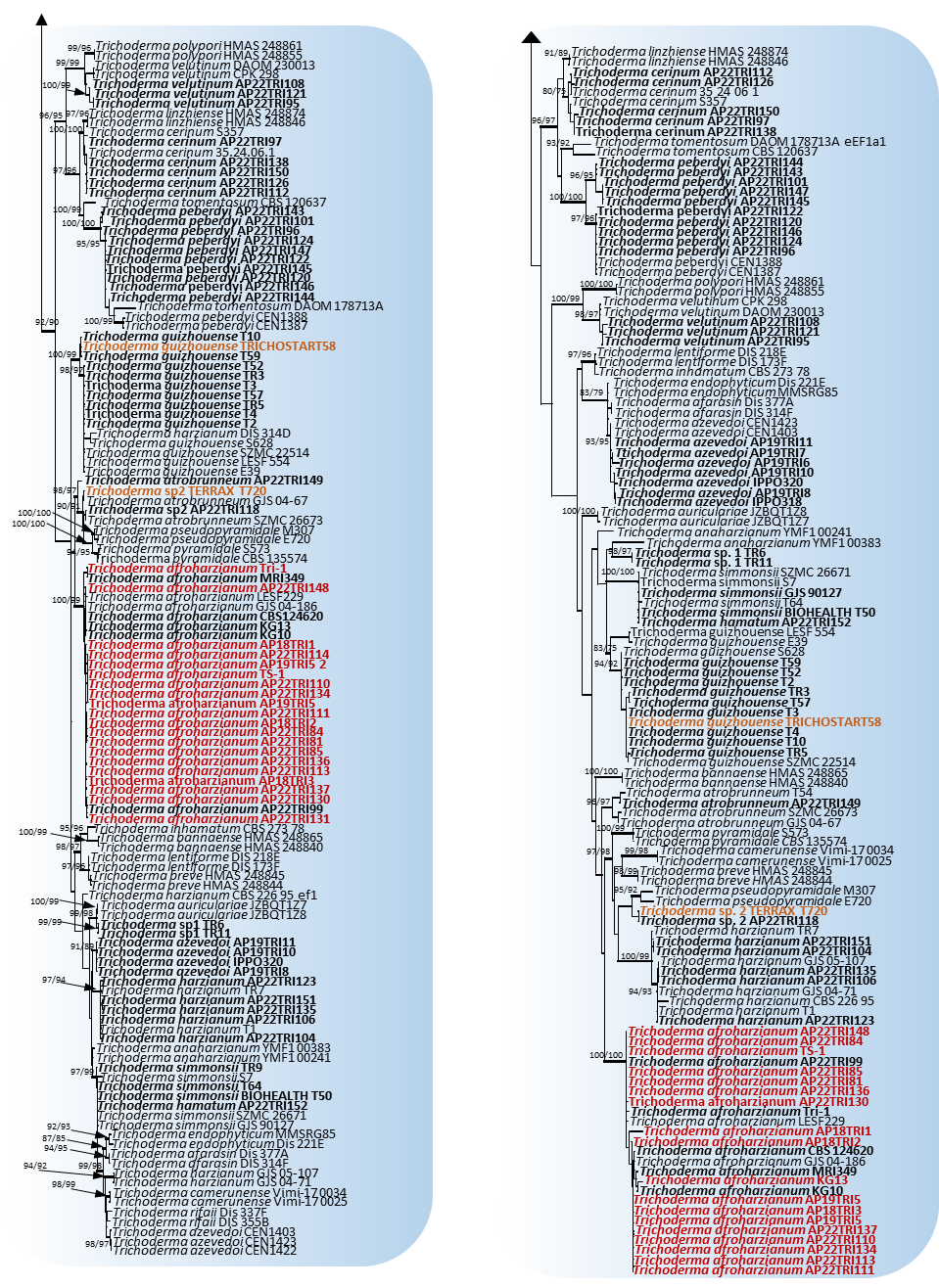


**A B**
